# Micro practices of coordination based on complex adaptive systems: user needs and strategies for coordinating public health in Denmark

**DOI:** 10.5334/ijic.1530

**Published:** 2015-09-29

**Authors:** Morten Deleuran Terkildsen, Inge Wittrup, Viola Burau

**Affiliations:** CFK – Public Health and Quality Improvement, Central Denmark Region/Interacting Minds Centre, Aarhus University, Aarhus, Denmark; CFK – Public Health and Quality Improvement, Central Denmark Region/Department of Public Health, Aarhus University, Aarhus, Denmark; CFK – Public Health and Quality Improvement, Central Denmark Region/Department of Political Science, Aarhus University, Aarhus, Denmark

**Keywords:** user needs-based coordination, complex adaptive systems, micro practices, public health, anthropological qualitative approach, Denmark

## Abstract

**Introduction:**

Many highly formalised approaches to coordination poorly fit public health and recent studies call for coordination based on complex adaptive systems. Our contribution is two-fold. Empirically, we focus on public health, and theoretically we build on the patient perspective and treat coordination as a process of contingent, two-level negotiations of user needs.

**Theory and Methods:**

The paper draws on the concept of user needs-based coordination and sees coordination as a process, whereby needs emerging from the life world of the user are made amenable to the health system through negotiations. The analysis is based on an explorative case study of a health promotion initiative in Denmark. It adopts an anthropological qualitative approach and uses a range of qualitative data.

**Results:**

The analysis identifies four strategies of coordination: the coordinator focusing on the individual user or on relations with other professionals; and the manager coaching the coordinator or providing structural support. Crucially, the coordination strategies by management remain weak as they do not directly relate to specific user needs.

**Discussion:**

In process of bottom-up negotiations user needs become blurred and this is especially a challenge for management. The study therefore calls for an increased focus on the level nature of negotiations to bridge the gap that currently weakens coordination strategies by management.

## Introduction

Health services are characterised by growing specialisation, for example as reflected in the concentration of certain treatments in particular hospitals, and this increases the risk of service fragmentation. Inter-organisational coordination has, thus, become a key focus in health systems, as reflected in the emergence of a plethora of care pathways which aim to standardise coordination in relation to major diseases such as breast cancer or diabetes [1, 2]. Care pathways presuppose clear clinical diagnoses, yet many health problems are highly complex [3]. This particularly applies to public health, which is characterised by public health problems that are difficult to define, that have no clear solution and that are both interdependent and multi-causal. For example, healthy food for pregnant women in a deprived area is as much a health problem as it is a social, economic and transport problem. Based on a recent study of public health partnerships in England, therefore, Hunter and colleagues [4] call for less formalised and strategic approaches to coordination based on complex adaptive systems. This is echoed by recent contributions to the literature on coordination more broadly [3, 5, 6], which suggest that the systems approach better fits the organisation of complex tasks like health services.

Correspondingly, there is emerging body of knowledge about the practice of this approach to coordination, where studies have analysed health service practice in different settings from the perspective of complex adaptive systems, including emerging departments [6] and local health networks [5]. We contribute to this literature in two ways. Empirically, the paper focuses on public health, where coordination is particularly complex; this makes for a particularly hard test for coordination based on complex adaptive systems. Theoretically, we conceptualise coordination as a process of negotiating user needs, which allows us to focus on the micro level practice of coordination. This is concerned with processes of turning user needs into public health interventions amenable to the health system. Our research question can be formulated as follows: what specific coordination strategies emerge from the negotiations between users and front-line coordinators, and between front-line coordinators and managers? The analysis is based on an explorative case study of a health promotion initiative in Central Denmark Region implemented in four municipalities. The initiative was chosen as it adopts user needs-based coordination; more specifically, it uses narrative health conversations, which start with the self-perceived (public) health problems and needs of users. The study adopts an anthropological qualitative approach [7] and draws on a range of data, including case files, blog postings, field notes and semi-structured interviews with front-line coordinators and their managers.

## Theory and methods

### Approaches to coordinating public health – from structures to user needs

There is an extensive health services literature that sees coordination as a problem of organisational structures and their management [8–12]. However, this sits uneasily with the broad nature of public health and its multiple interdependencies [7]. Public health goals typically lack clarity and are often contested. Similarly, public health functions tend to be broad and encompass health promotion and health prevention as well as health service improvement. Hence, public health problems are typically characterised by a high level of complexity: they are difficult to define, they have no clear solution and they are both interdependent and multi-causal [7, 13]. As a result, public health problems typically transcend existing organisational and professional boundaries, thus making coordination difficult. At the level of the individual health professional this has two consequences: a high number of very different partners and many interactions [14].

Significantly, there is little evidence that coordination initiatives to date have led to better public health outcomes [15]. This is confirmed by recent studies from other health care settings. For example, based on their analysis of local health networks in Ontario, Tsasis and colleagues [5] conclude that one possible explanation for the lack of organisational change towards integration is that the health system continues to be treated in a linear fashion, specifically reflecting the reductionism and determinism associated with the predominant scientific management paradigm. Similarly, in a more conceptual paper Edgren [3] argues that the machine metaphor has long shaped the view of an effective organisation in health services. However, this view is inappropriate considering the changing needs and preferences of patients.

Against this background, recent contributions to the literature suggest moving towards less formal and less strategic approaches to coordination based on complex adaptive systems [3, 5, 6, 16, 17]. This involves seeing tasks, like the coordination of public health, in a more holistic way as whole systems rather than discrete concerns. Such systems are complex, as the ability to undertake tasks does not rest with one single organisation, but instead requires coordination across a range of organisations. Here, systems typically proceed in an adaptive manner and approach tasks in successive waves of interaction. More specifically, complex adaptive systems have a number of key characteristics [3, 18]: they have fuzzy boundaries, are embedded in one another and co-evolve, and they have natural tensions that cannot necessarily be resolved, and they are unpredictable.

The concept of complex adaptive systems has been criticised for being too abstract and for lacking clarity in terms of consequences for practice [3]. Against this background, we find Seddon's [16] work very helpful as he clearly spells out the implications for organising health services. He argues that applying complex adaptive systems to organisational change means trying out multiple approaches which directly arise from what works best in practice. This requires integrating decision making into work flows and by extension designing services based on user needs. For example, this involves delegating needs assessment to front-line professionals and approaching needs assessment in a flexible way that can accommodate the concrete and potentially changing needs of users. For management this implies developing strategies for integrating user needs both internally between front-line staff and management and externally building partnerships with relevant service providers. For example, management needs to insist on the primacy of individual user cases and treat this as a springboard for supporting professionals in their daily (coordination) practice and for establishing connections across sectors. In short, this calls for moving beyond the macro level perspective on coordination inherent in the systems approach and instead towards a focus on the micro practices of coordination.

As the anthropological literature on health care interactions suggests, such micro practices of coordination based on user needs are likely to consist of negotiations [19–21]. Interactions in health care are not linear transactions of information from one actor to another, but instead are highly complex, situated negotiations. As patients enter the health care sector they negotiate their (public) health needs with health care professionals who play a key mediating role as coordinators, negotiating further actions with managers. In the process, user needs emerging from the life world of the user are gradually made amenable to the infrastructure of the health system.

### Methods for studying micro practices of coordination based on user needs

We analyse the micro practice of coordination based on user needs as a two-fold process of negotiation: first, between the front-line coordinator and the user, and second, when front-line coordinators seek managerial support. Our analysis is based on a case study of a health promotion initiative in Central Denmark Region. The initiative was chosen as it adopts user needs-based coordination; more specifically, it uses narrative health conversations, which start with the self-perceived (public) health problems and needs of users. The initiative was implemented at the same time in four municipalities and the overall aim was to reduce health inequalities among users from ethnic minorities and to better understand the complexities of social life in migrant families [22]. The four municipalities were comparable in terms of population size and percentage of non-Western migrants; two municipalities were more urban, while the other two were more rural. While the field of public health spans broadly, since a major reform in 2007, municipalities in Denmark are responsible for implementing primary public health promotion and prevention initiatives. Here the municipalities work closely with the country's five regions that run hospital services.

As part of the present initiative, each municipality appointed a front-line coordinator who was responsible for facilitating the coordination of services on a case-by-case basis. Three coordinators were nurses by training, while one had a master's degree in public health. Each municipality also appointed a manager responsible for integrating the coordinator into the municipal health system. Three of the managers had a background in nursing, while one had a degree in political science.

The coordinator conducted an initial conversation with the user to help define individual needs and subsequently contacted relevant organisations to jointly put together a plan of activities. The initial conversation was based on a narrative approach, departing from the idea that there is a connection between the telling of stories and the positioning and construction of meaning in individual lives [23, 24]. The narrative conversation itself used questions such as “Where do you see yourself in three years?” and “How does your health relate to where you want to be in three years?” as well as questions concerned with the present “Tell me about your daily life” and the past “How did you come to Denmark”; the conversation thus situated users in an explicit “life story timeframe”, helping them to connect aspirations for their future health with their present life situation and experiences from the past [25]. At the end of each conversation, the coordinator and the user decided what public health interventions could be help realise the future the user envisages.

This reveals not only hidden cultural values and norms, but also individual perceptions of health and (public) health problems to the coordinator. The coordinator conducted another narrative conversation after the completion of the activities listed in the plan and again after nine months. These follow-up narrative conversations focused both on evaluating the activities and on what has happened with the life of the user in general.

Data were collected over a period of three years (2010–2013) by members of the research team, which consisted of two anthropologists and one political scientist. Data came in three forms. First, the case files (130 in total) written by the front-line coordinators, covering the three narrative conversations with each user and any follow-up activities. The level of detail of the files differed. While some coordinators would only write a summary of the narrative conversation, others would include direct quotes from the patients. Second, the information shared by the network of coordinators and the project management on a secure online blog. Third, field notes from participant observations written by first author. Throughout the project period he participated in network meetings with the coordinators, where individual cases were discussed.

In terms of case selection, we first identified two user cases in each municipality. This would allow us to conduct an in-depth analysis of the collected material, while maintaining an overview of the challenges of the mirco practice of coordination. The case selection was theoretically guided and we chose eight cases that represented a particularly complex set of (public) health problems, where users drew from a wide range of everyday life positions, and where health thus became intertwined with economic, social and mental health problems. The participants in the case selected came from Bosnia, Iran, Somalia, Sri Lanka, Thailand and Turkey, and included five women and three men. Their ages ranged from 30s to late 50s.

In order to detect significant issues concerning the practice of coordination, all three authors read, discussed and compared the combined the different types of data focusing on three issues: the central problems of the user as identified by front-line coordinator, the activities initiated by the coordinator and the dilemmas encountered in the process. On the basis of these themes we designed a guide for semi-structured interviews with the four coordinators. As our analysis below shows, coordination activities involved drawing on other professionals, but we decided to focus on interviewing managers to gain insights into the limitations of horizontal coordination among different professionals. The interviews with the manager heading the unit of the respective coordinator included questions about the practice of supporting coordination through leadership and the understanding of the coordination specifically as related to users from ethnic minorities.

The four interviews with the front-line coordinators and the four interviews with their managers were conducted by last author in 2011/2012 and lasted between 30 and 60 minutes each. All interviews were transcribed verbatim and analysed with the software programme NVivo. Each case and interview quoted in this article is anonymous as agreed with the respondents. In relation to the analysis of the interview material, we followed an anthropological qualitative approach outlined by Hammersley and Atkinson [7] as well as Spradley [26] that focuses on narrowing data, through multiple readings of the material while gradually building and testing empirical concepts against a theoretical frame. According to Wadel [27], this approach of moving back and forth between the theoretical concepts and the empirical material in a “round-dance” ensures that both theory and data are subjected to continued reflection in the process of the analysis. In practice, we read the interviews both individually and as a group through several rounds, starting with an initial overview, followed by a second round focusing on the structure underlying the interviews and on identifying initial concepts, and a final more interpretative reading developing general typologies out of the case material.

## Results: strategies for needs-based coordination in practice

### Negotiations between coordinators and users

In the context of the narrative conversations, coordinators continuously walk a fine line between exploring the individual narrative on the one hand, and making choices about which (public) health problems to explore further on the other. As part of the negotiations with the user, the strategy for coordination typically crystallizes at the end of the conversation, when the coordinator outlines her assessment of the user's (public) health problems and needs. From the case material, two overlapping strategies emerge: coordination with the purpose of “creating inner order” and “creating outer order”.

#### Creating inner order

All coordinators are deeply concerned with creating a situation of comfort and trustworthiness during their conversations with users. The coordinators focus on creating opportunities for the user to reflect on her/his situation and on supporting users in this process. One coordinator describes this as follows.

I sum up what has been said … I ask him what he needs… In addition, I am very aware to ensure true dialogue between equals, and not to be controlling when the conversation takes another turn! (Coordinator 2)

This points to an approach characterised by open-mindedness with a focus on creating equality and signifies a deeply rooted commitment among the coordinators to expressing empathy.

Central strategies of the coordinator include praise, acknowledgement and respect of the user and her/his storyline. Through this, the coordinator tries to strengthen the user's capacities to pursue new goals in life and to reassure him/her that help is available if needed. The underlying rationale is to instil in the user a feeling of “being-seen-and-being-heard”.

Another coordinator goes further and stresses the quality that lies in the conversation itself and claims that: “Words help by themselves” (Coordinator 1). This suggests that the narrative conversation has the potential of becoming a goal in itself. In such crucial moments the coordinator engages in a process of thoroughly understanding and supporting the reflections emerging as part of the narrative of the user; here, the conversation is ascribed a healing potential.

Sometimes coordinators expand their professional competencies by bringing in personal experiences during the conversation as is the case in the following example:

From the very beginning my role in this case primarily has been to be a person whom [the user] could trust. What she really needs is a friend. She needs someone who is there. Someone she can call. (Coordinator 4)

Being invited into the very intimate sphere of private lives represents a challenging moment for the coordinator. On the one hand, the invitation indicates that the user is very happy with the coordinator's efforts and, on the other hand, indicates a loneliness in the life of the user. Furthermore, the invitation challenges the coordinator's level of involvement and forces her to draw a line, which could lead to an awkward situation.

In connection with the dilemma outlined above, the coordinators pick and choose amongst a great variety of themes including: family problems such as the role as husband, wife, mother or father; the gossip in local ethnic minority community; sexuality; or reflections on how it is to live life in a native Danish family.

In sum, the main rationale underlying the strategy of “creating inner order” is to activate the resources of the users to look how to address their health need. The underlying mechanism is that the coordinators acknowledge the deep frustration of living with complex (public) health problems and help to develop a sense of cohesion by taking part in the user's reflections. In some cases, the very fact of creating inner order is just what is needed, whereas in others this must be supplemented by services/activities from other parts of the health system in the broadest sense.

#### Creating outer order

Many users with complex health needs meet the coordinator when they have been sent back and forth within the system many times; the following case is indicative:

We all need to be taken by the hand sometimes … this guy [name of the user] is very interested in physical activity. But he does not go to the general practitioner to get a referral. He can't go to the rehabilitation department either, because he does not have a rehabilitation plan from the hospital. In fact, he does not have any plan at all. That is, what I can do [put a plan together]. (Coordinator 3)

This confusion points to the fact that many different provider organisations are involved and for the user it becomes difficult to choose which provider to approach with a view to further treatment. Such situations confront the coordinator with many loose ends of potential services and very often it is her role to cut through those barriers by sorting out the papers and putting together a package of services/activities.

In the case files the coordinator, therefore, typically summarises the narrative conversation by listing what to do next, as the following example illustrates:

We agree that I can contact the general practitioner,… [and] the caseworker … in order to inform [her] about our conversation today and if possible to gain more knowledge about where [the user] is in the clarification process…. That was the underlying idea. Because this was in no way evident for the user. (Coordinator 1)

From the case notes, the role of the coordinator emerges as a “bridge builder” who tries to gather and translate information from other institutional worlds for the user who is not able to do that alone. Doctors, caseworkers, lawyers are typical examples of external contacts, but very often the coordinator may also contact swimming pools, gyms, charity shops, chiropodists, dieticians and family counsellors.

Nevertheless, the strategy of “creating outer order” also has a strong enabling and empowering element, where the coordinator sees herself as supporting a process, whereby the user gradually takes hold of the messiness of his/her situation and tries to create some order; “…get a handle on his/her situation” (Coordinator 1), as the coordinator says. This may also require not standing in the way of users:

We [the coordinators] are there to support and get people to get on with their lives, and when they are on their way we withdraw…. Because I also have to keep a distance between us [the coordinator and the user] … because if not then [the user] could swallow me completely! (Coordinator 1)

The coordinator connects the health needs of the user to the outer world in order to create new opportunities for the user. For a time, the coordinator takes charge of the user's life, only to leave her to her own devices again. This can be a critical moment, where the coordinator must cut ties and, at the same time, make sure that the user is able to continue on her own, so that the coordinator can move on to the next user.

The practice of creating outer order and negotiating with other professionals from both inside and outside the health system is characterised by cultural and legal challenges. One coordinator has a long series of interactions with a user who had lost a baby in her native country. The user agrees to visit a psychologist to assess if she is suffering from depression.

It was an incredibly bad experience for her [the user] to see that psychologist. First of all, she was referred to a man. Something one should never do with a woman with an ethnic minority background! That does not work at all… He [the psychologist] diagnoses a depression and she gets some medicine, which gives her a lot of side effects. She was completely thrown off. (Coordinator 4)

The coordinator gets caught in a misreading of what is cultural and what is psychological and positions herself on the side of the user. In order to get out of the dilemma, she then turns to the social worker to help the woman arrange a holiday her native country, as the coordinator considers this a good opportunity for recovery. However, this initiative is marred with legal problems:

But then we came up against the rules of the benefits department, which regulate if a user can receive social security [benefits] when on holiday. Those rules had just been tightened up. And our office in the municipality is not very open to negotiations. So she [the user] had to leave [for her holiday] without any money. (Coordinator 3)

Coordinating complex health needs in a highly fragmented health system at times puts the coordinators in extremely difficult situations. No matter where they turn, there is little room for manoeuvre. For those issues that emerge at the front-line level, but which cannot be resolved by the coordinator herself, the coordinators become highly dependent on management to work across sectors. Therefore, the negotiations between the coordinator and the line manager are the other level at which strategies for managing coordination emerge.

### Negotiations between coordinators and managers

From the interview material across the four municipalities, two distinct strategies for needs-based coordination emerge from the negotiations between front-line coordinators and their management. We have chosen to call these “coaching management” and “structural management”.

#### Coaching management

All managers are perfectly aware of the challenges of providing public health for users with ethnic minority backgrounds. The managers are highly conscious of the blind spots of their organisation and that it needs to take more explicit steps to deal with the special needs of such users. The managers, therefore, pay close attention to the particular work situation of the coordinator. Among other things, they all mention the pitfall of loneliness in the job, which the managers try to address in different ways.

In the interviews, the managers express great respect for the work of the coordinators and for how they approach their job. One manager formulated this in the following way:

Sometimes she [the coordinator] says to me: shall I go on or is it too much?… I don't have the answer, either, you see. That's when I say, if you feel that it's necessary then call [me] again. (Manager 3)

This points to an “open door approach” where the coordinator can take any issue of concern to the manager. Such issues are often emotional and ethical in nature, challenging the coordinator on a personal level in her day-to-day work. The coordinator may expect some advice in relation to her issue of concern, but the manager is clear about not giving any advice. In her view, the manager has neither the authority nor the knowledge to give “right” answers. Instead, the manager expresses confidence in the coordinator's skills and her ability to make appropriate choices, offering only general comforting assurance and expressing her general trust in her decisions. Yet, this trust provides a broad shoulder for the coordinator to lean on.

This type of “managing by coaching” is confirmed by another manager who sees her role as offering support and acknowledgement:

But because I can coach her [the coordinator] and raise some questions when she [the coordinator] has problems, and that is pretty much what I do… I think, she [the coordinator] very much needs to know from me, that what she is doing is ok! (Manager 2)

The coaching relationship, however, is not only for the benefit of the coordinator. The coaching activities also help the manager understand the personal struggles and frustrations resulting from working with complex (public) health problems.

She [the coordinator] really needed to talk her things through … That is fine and then I think it's exciting to listen to the experiences she [the coordinator] has with it [her work]. What is difficult and what is easier. (Manager2)

Although individual user cases are explicitly discussed during meetings between the manager and the coordinator, the focus is not on the user as such. Instead, the coordinator personally and the challenges the coordinator encounters are the main concerns. This makes coaching at the same time personal and managerial.

#### Structural management

The other strategy of needs-based coordination emerging from the negotiations between coordinators and managers focuses not on coaching but on structure. As one manager puts it:

What [strategy] should we pursue? [I feel:] … define the lines, but not [have] … detailed discussion[s].… There have been issues like: How can we organise funding issues related to the coordinating activities and what kind of procedures need to be introduced in this respect? And what kind of stuff should we report on. (Manager 4)

Structural management strategies are centred around issues of funding and the organisation itself. Here, management aims to shield the coordinator from structural issues concerning the overall organisation, so that she can focus on her front-line work without the manager trespassing the “specific professional world” of the coordinator. As one manager comments:

I think we can't do it [providing public health for users with ethnic minorities] without having extra resources attached. So those are the concerns that I … have. Whereas the coordinator of course also has the more professional considerations. (Manager 2)

The interviews also show that, although there is considerable acknowledgement of the complexity health needs, managers explicitly separate the particular the world of user the coordinator deals with from the managerial perspective of seeing the organisation and its structure as a whole. As one manager remarks:

I'm in that way not a part of it [the world of the user]. So … what I'm telling you comes from a management perspective, and that has to do with working in an organizational way … What I think I can contribute … most of all is to stick to the fact that … we are dealing with a project, where we have to gain knowledge, [and which we have] to unfold and implement…. (Manager 1)

In the quote, the manager acknowledges that there is a vertical communication gap contained in the distinction between the world of the user (and by extension the coordinator) and the organisational perspective of the manager. This, despite the fact that the managers in all municipalities try to create vertical communication structures with the coordinators in varying ways.

Two municipalities have a “direct, vertical coordinator-to-manager communication strategy”. Here coordinators and managers meet both formally and ad hoc to discuss challenges relating to the project as a whole and the future strategies of implementation and development. In one of the municipalities, the two even share an office. Another municipality is organised vertically around a team structure:

The coordinator here is part of a team. That is something that we, definitely I, stress, very much in my approach to management; that you [the coordinator] are part of a team. (Manager 1)

In this case, the team is responsible for assisting the coordinator in the day-to-day challenges of working with the particular world of the user, whereas management is brought in when decisions regarding overall strategies are on the agenda. The health department has also formalised the collaboration with the employment department.

Lastly, one municipality has a two-fold vertical management structure, where a middle manager sits in-between the coordinator and the senior manager to offer day-to-day, hands-on assistance in dealing with specific challenges that arise from individual users. Also here, meetings between coordinator and the senior and the middle manager become an arena for discussing overall strategies and future areas for development. Irrespective of the specific approach, the meetings between manager and coordinator all have a common feature; they concerned with overall strategies rather than individual cases. The focus is on “the whole” rather than “the particulars”.

## Discussion

The aim of the article was to contribute to an emerging body of knowledge about the practice of coordination based on complex adaptive systems. Empirically, we focus on public health and, while theoretically, we conceptualise coordination as a process of negotiating user needs and thereby highlight the micro practices of coordination. User needs emerge from the intertwining problems in the life of users. These problems go well beyond medical notions of health and they are constantly in flux. The micro practice of coordination emerges from the negotiations among users, coordinators and managers, which turn user needs into public health interventions amenable to the health system.

There are multiple and rich bodies of literature focusing on the clinical practice of coordination and how to better integrate the views and experiences of users in health care decision making [28–30]. The literature introduces a great variety of approaches, including: case management [31, 32], holistic needs assessment [33, 34], and co-produced care plans [35, 36]. The literature is particularly concerned with specific instruments and their effects. Studies emphasise the user perspective and suggest that coordination based on distinct models and micro practices is likely to be most effective. We build on this literature by acknowledging the primacy of the user perspective; but we aim to embrace complexity by treating coordination as a process of contingent, two-level negotiations among professionals and with management [37]. As the analysis shows, this translates into a two-fold challenge for the front-line coordinator. First, the challenge is, together with the user, to prioritise her/his needs, paying specific attention to the particular world of the user, but simultaneously transforming user needs into a viable and relevant package of services/activities. The subsequent challenge, second, is to further carry these users’ needs into the health care sector, by negotiating them with managers when seeking support for daily coordination activities.

Looking at the experiences of front-line coordinators and their managers, the analysis identifies four strategies that span across different directions of coordination (horizontal and vertical) and across different types of focuses (internal and external). Table 1 offers an overview of the strategies.

The front-line coordinators use two types of strategies of horizontal coordination. The strategy of “creating inner order” is concerned with giving the user opportunities to reflect on her life through the narrative conversations to, not only, strengthen the trust between the two, but also help the user to live with her illness. Therefore, the narrative conversation becomes a goal in itself, whereas in the strategy of “creating outer order” the narrative conversion is merely a means to connect the user with other (health) services and professionals. The bridge building occurs in two steps: the front-line coordinator gathers and translates relevant information for the user and through this allows the user to gradually become able to take charge of her own life. This involves negotiating relevant (health) services/activities with other professionals/providers although, in the process, the front-line coordinators typically meet considerable legal and cultural challenges. The front-line coordinators, therefore, become dependent on management understanding and acting on the specific health needs of users, for example, by managers negotiating with their counterparts in other services (horizontal coordination at management level).

The managerial response is two-fold. The strategy of “coaching management” first and foremost pays attention to the specific work situation of the front-line coordinator and any emotional and ethical concerns that might arise. The manager offers the coordinator general support and assurance, instead of substantive advice in relation to the particular user case at hand. In contrast, the strategy of “structural management” primarily deals with overall issues of funding and organisation. Here the underlying idea is to protect the front-line coordinator from such issues. Importantly, the strategy rests on a separation between the world of the user/front-line coordinator on the one hand and the world of the manager/organisation on the other hand.

Yet looking more closely at our analysis, although the strategies employed by the managers have a vertical direction, they do not directly relate to the specific cases of individual users the front-line coordinator is confronted with. The absence of important vertical links has been found by other studies [17, 38]. However, our understanding of needs-based coordination as a process of negotiating user needs allows us deeper insights into where the crucial link goes missing.

In the negotiations which occur when managers support front-line coordination, the emerging user needs leave aside the focus on the particular user. Neither coaching management nor structural management relate directly to the user needs negotiated between the user and coordinator as part of the initial narrative conversation and the later meetings. Instead, the analysis shows that coordination strategies are built on negotiated user needs that are directed either at the individual coordinator or at generic structures of the organisation. This makes the vertical link of coordination between the front-line coordinator and the manager rather weak. From a theoretical perspective, it becomes evident that during the two-level process of negotiating user needs, the particular world of the user becomes gradually diluted as user needs are negotiated from the bottom-up. This in turn leads to a gap between managers and the user needs negotiated with coordinators on the one hand and the user needs negotiated between front-line coordinators and users. Importantly, this disconnect undermines the possibilities for a truly parallel horizontal coordination at both front-line and managerial levels.

## Conclusion

The article started by suggesting that approaching coordination as a question of organisational structures and management is problematic in the case of public health, which is characterised by highly complex (public) health problems. Instead, we follow recent contributions to the literature on coordination, which make a case for an approach based on complex adaptive systems. We conceptualise the practice of coordination as a contingent and two-level process of negotiating user needs and set focus on the micro level, using a health promotion initiative in Central Denmark Region as a case study. The initiative operationalises the needs-based focus by means of a narrative health conversation, which starts with the world of users and their self-perceived (public) health problems and needs.

However, as our analysis has shown, adopting an approach to coordination based on complex adaptive systems has its own challenges, especially relating to management. In contrast to coordinators, managers seem to continue to adhere to the assumptions of a health system based on certainty and specialisation, and vertical strategies of coordination tend to focus on reducing complexity by managing either personnel or structures. In doing so, strategies miss their target, namely coordinating of complex health needs, which above all are always highly particular, as they are rooted in worlds of individual users. This raises a number of more general points. First, the scientific management paradigm is hard to abandon, even in initiatives like the present that explicitly set out to approach coordination based on complex adaptive systems. Also here, the reductionism and determinism associated with scientific management turn out as an important barrier for coordination [5]. Instead, a type of management is required that draws on multiple and incremental approaches. As Grint [13] stresses, managers must try new things and learn to operate in a clumsy rather than a perfect solution environment. Second, even where complex adaptive systems have a strong self-organising capacity, they require support that is direct and specific, as the practice of front-line coordinators in the present initiative powerfully illustrates. This challenges the notion in the literature that support should best be indirect and hands-off, offering “direction without directiveness” [3, 5]. Here the underlying idea is that self-organisation is about creating order without the help from the outside.

With its focus on complex practices of coordination, this article offers a deeper understanding of the origins and substance of the challenges managers encounter. The challenges are connected to the process of negotiating user needs and turning them into public health interventions that are amenable to both, the world of the user and the health care system. This calls for an increased focus on the two-fold process of negotiations, in order to bridge the gap that currently weakens vertical coordination strategies.

## Figures and Tables

**Table 1.**
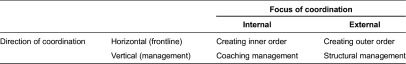
Strategies for coordinating intersectional (public) health problems within the narrative approach
